# From ecology to engineering: the role of myxobacteria in recirculating aquaculture systems

**DOI:** 10.1128/aem.01376-25

**Published:** 2025-08-27

**Authors:** Oskar Modin

**Affiliations:** 1 Division of Water Environment Technology, Department of Architecture and Civil Engineering, Chalmers University of Technology 11248, Gothenburg, Sweden; Michigan State University, East Lansing, Michigan, USA

**Keywords:** myxobacteria, geosmin, recirculating aquaculture systems, microbial ecology, off-flavor, environmental biotechnology

## Abstract

Open microbial communities play vital roles in many engineered systems, providing essential ecosystem services but also posing operational challenges. In recirculating aquaculture systems (RASs), microbial activity is crucial for water purification, yet it can also lead to the accumulation of taste-and-odor compounds that compromise fish quality. In a recent study, Södergren et al. (Appl Environ Microbiol 91:e00757-25, 2025, https://doi.org/10.1128/aem.00757-25) report the first successful isolation of myxobacteria from RAS and demonstrate their ability to produce geosmin and other volatile organic compounds under various nutrient conditions, including in real RAS water. This work provides foundational insights into the ecological roles of myxobacteria and their contributions to off-flavor formation in aquaculture environments. In this commentary, I reflect on the broader significance of microbial ecology in environmental biotechnology and discuss how the findings of Södergren et al. may inform future strategies for managing microbial communities in RAS to improve system performance and product quality.

## COMMENTARY

**Fig 1:**
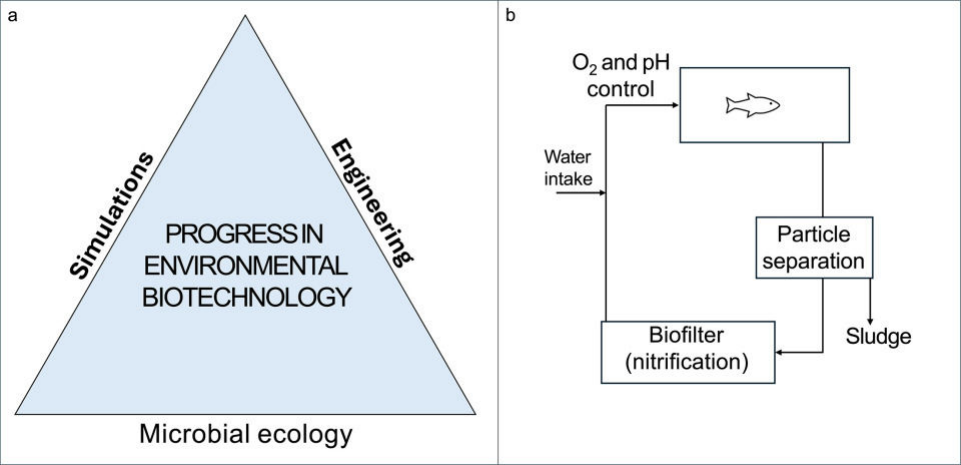
(**a**) Conceptual model of progress in environmental biotechnology as a combination of microbial ecology, simulations, and engineering. (**b**) An example of a schematic of a recirculating aquaculture system. Denitrification may also be included for complete nitrogen removal. Another alternative is aquaponics systems, where plants utilize nutrients in the circulating water.

Environmental biotechnology relies on the management of open microbial communities to deliver services such as water and wastewater treatment, pollutant degradation, energy and food production, and public health protection (1). I propose that advances in environmental biotechnology can be illustrated by a conceptual model comprising three interlinked components: microbial ecology, simulations, and engineering (Fig. 1a). Microbial ecology provides the foundational understanding of microbial interactions and functions (2). This knowledge enables the development of models that simulate microbial community responses to environmental conditions. These simulations, in turn, guide the engineering of systems that harness microbial processes for specific outcomes, such as water purification in recirculating aquaculture system (RAS). The cycle is iterative: insights from engineered systems feed back into ecological understanding, refining both models and designs. This integrated approach has driven significant advances, for example, new and improved technologies for nitrogen removal from wastewater and valorization of organic waste streams (3, 4).

RAS is a technological system for food production in which open microbial communities play essential roles. By continuously recirculating water, the system enables fish farming with minimal water consumption (Fig. 1b). However, fish feces contaminate the water, necessitating microbial processes for purification. Nitrification is particularly critical for reducing ammonia toxicity (5). While microorganisms are vital for maintaining water quality, they can also pose challenges. Pathogenic microbes may cause disease outbreaks (6), and certain microbial metabolites, referred to as taste-and-odor compounds or off-flavors, can accumulate in fish tissue and render it unpalatable (7). A common mitigation strategy is depuration in clean water for several days to weeks while depriving the fish of food. This consumes large volumes of clean water and leads to weight loss in the fish (8). Consequently, depuration has economic implications for fish farmers, reduces RAS efficiency due to high water consumption and lower product yield, and may negatively impact fish welfare through stress associated with handling and starvation. With global fish demand projected to double by 2050, and aquaculture expected to meet this need, RAS is poised to grow (9). A deeper understanding of the underlying causes of taste-and-odor issues and the development of effective mitigation strategies would benefit the industry.

Geosmin and 2-methylisoborneol (2-MIB) are widely recognized as the primary odor-producing compounds in RAS. These secondary metabolites are produced by various bacteria and some eukaryotes and are also known to cause taste and odor issues in drinking water (10). While bacteria from the phyla *Cyanobacteriota*, *Actinobacteriota*, and *Myxococcota* have been identified as geosmin producers, recent studies employing amplicon sequencing of the geosmin synthase gene and genome-resolved metagenomics suggest that the diversity of geosmin-producing organisms is likely broader than previously recognized (11, 12). The photosynthetic cyanobacteria are mainly present in outdoor systems, whereas actinobacteria and myxobacteria are associated with indoor RAS (7, 8). Members of the genera *Streptomyces* and *Nocardia* within *Actinobacteriota* have been isolated from RAS and shown to produce geosmin (13, 14). However, culture-independent methods have shown that both actinobacteria and myxobacteria can be abundant (12, 15, 16). Although myxobacteria have previously been isolated from environments such as soil (17), Södergren et al. (18) are the first to successfully isolate them from RAS and quantify their production of geosmin and other taste-and-odor compounds.

Their study offers several key contributions that could advance microbial management in RAS. First, the authors quantify geosmin production per cell under varying nutrient conditions. This is an essential step toward developing mathematical models that can predict off-flavor concentrations in RAS water. While a model for the uptake and elimination of geosmin and 2-MIB in fish flesh has been proposed (19), there is a notable gap in models that incorporate microbial production of off-flavors within biofilters and other system components. Although dynamic models exist for water quality parameters such as organic carbon and nitrogen (20), integrating taste-and-odor dynamics requires detailed knowledge of the microbial producers, the conditions under which they operate, and their production rates. Södergren et al. provide initial data to support such a development.

Second, a major strength of the study lies in its comprehensive quantification of volatile organic compounds. Using gas chromatography coupled with mass spectrometry and olfactometry, supported by a sensory panel, the authors identified a wide spectrum of both known and previously uncharacterized compounds produced by the myxobacterial isolates. While geosmin and 2-MIB are well-established contributors to the “earthy” and “muddy” off-flavors in RAS, other volatile organic compounds may also be relevant (21). This broader chemical profiling is crucial for engineering better systems, as it informs which compounds should be monitored and mitigated.

Third, the study contributes to our understanding of microbial ecology in RAS by exploring the ecological roles of the isolated myxobacteria. The authors isolated 16 additional heterotrophic strains from RAS water and demonstrated that the myxobacterial isolates preyed on 14 or 15 of these strains. Interestingly, neither isolate could prey on a *Tahibacter* sp., suggesting that predatory interactions may shape microbial community structure by selectively targeting specific taxa. Current process models, such as those describing nitrification, often represent microbial decay as a simple first-order process (22). A better understanding of microbial predation could lead to improved representations of decay in process models and, ultimately, more accurate reactor designs. The findings of Södergren et al. (18) represent a step in that direction.

The isolation and characterization of two myxobacterial strains, *Myxococcus virescens* AT3 and *Corallococcus exiguus* AT4, from RAS represents an advancement in understanding the ecological role of myxobacteria and their contribution to taste-and-odor issues in these systems. However, as the authors themselves acknowledge, there is often a disconnect between the fast-growing strains typically isolated in laboratory settings and those that dominate complex microbial communities *in situ*. This underscores the importance of combining cultivation-independent approaches, which can reveal the relative abundance and distribution of taxa in real systems, with cultivation-based methods that provide mechanistic insights into the conditions under which specific taxa produce off-flavors. The term “reverse metagenomics” was used by Podar and Reysenbach (23) to describe how cultivation strategies can be informed by metagenomic data on the genetic potential of target organisms. Perhaps such an approach could be used to culture putative off-flavor producers identified in metagenomic studies (e.g., references 12, 24). In a sense, this approach was also reflected in the present study. Previous culture-independent investigations had already indicated the ecological relevance of myxobacteria in RAS. These findings motivated Södergren et al. (18) to pursue targeted isolation and characterization of myxobacteria from RAS environments. Their results are essential for elucidating the functional roles of these organisms within RAS microbial ecosystems and for informing future efforts to model and manage taste-and-odor dynamics. Microbial management strategies have previously been proposed to reduce the risk of pathogen invasions in RAS (25). Perhaps microbial communities could also be managed to mitigate off-flavor issues.
